# Effect of Rare, Locally Isolated Entomopathogenic Fungi on the Survival of *Bactrocera oleae* Pupae in Laboratory Soil Conditions

**DOI:** 10.3390/microorganisms13040811

**Published:** 2025-04-02

**Authors:** Spiridon Mantzoukas, Alexandros Margaritis, Chrysanthi Zarmakoupi, Vasileios Papantzikos, Thomais Sourouni, Vasiliki Georgopoulou, Panagiotis A. Eliopoulos, Ioannis Lagogiannis, George Patakioutas

**Affiliations:** 1 Institute of Mediterranean Forest Ecosystems, Terma Alkmanos, 11528 Zografou, Greece; 2 Department of Agriculture, University of Ioannina, Arta Campus, 47100 Arta, Greece; alexmarg257@gmail.com (A.M.); chris.zarm@hotmail.com (C.Z.); b.papantzikos@uoi.gr (V.P.); tsourouny@gmail.com (T.S.); basilikh.georgopoulou5@gmail.com (V.G.); gpatakiu@uoi.gr (G.P.); 3 Laboratory of Plant Health Management, Department of Agrotechnology, University of Thessaly, Geopolis, 41500 Larissa, Greece; eliopoulos@uth.gr; 4 ELGO-Demeter, Plant Protection Division of Patras, NEO & L. Amerikis, 26444 Patras, Greece; lagoipp@gmail.com

**Keywords:** survival time, EPF, local strains, mortality, *Bactrocera oleae*, *Aspergillus*, *Fusarium*, *Lecanicillium*

## Abstract

Greece’s olive oil production is significantly affected by the olive fruit fly *Bactrocera oleae* (Diptera: Tephritidae), and its presence is perceived when it is too late to act for damage recovery. In this work, some unexplored entomopathogenic fungi (EPFs) were studied for their efficacy on olive fruit fly pupae in soil samples. Olive grove soil samples were collected to evaluate the effect of EPFs in their natural environment. The parameters that were analyzed to evaluate the performance of EPFs on *B. oleae* included the adult survival time, pupa hatch time, and the presence of mycelium on *B. oleae* pupae and dead adults. The efficacy of some EPFs was highlighted by the mycelium present on dead *B. oleae* adults after treating pupae with fungal isolates on the soil substrate. The results showed that for the soil substrate, external fungal growth was observed in dead adults with *A. contaminans*, *A. keveii*, *A. flavus P. lilacinum*, and *T. annesophieae* (100%). Remarkably, the lowest male proportion for soil and non-soil substrates was for *A.* flavus (0.41–0.42) for the first time, for *A. keveii* (0.36), and for *P. citreosulfuratum* (0.41) on the soil-only substrate in contrast to the control treatment (0.5 for both substrates). Given the high infestation caused by the olive fruit flies in Greece, the results of the study emphasize to use of incorporating certain EPF-based biopesticides into integrated pest management (IPM) programs.

## 1. Introduction

Olive cultivation is crucial for the economy of Mediterranean countries [1,2], as more than 70% of the world’s cultivation is in this region, reaching 4.3 million ha in Spain, Italy, and Greece [3]. Greece ranks as the third-highest producer country worldwide, with an average annual production of 300,00 Mg of olive oil, and the export of its olive products is a profitable source of income for Greek farmers [4]. Many studies have shown that Greek table oil is exceptionally nutrient rich [5], with approximately 16% of the annual production worldwide. However, damage from *B. oleae* (Diptera: Tephritidae) in some areas of Italy and Greece resulted in a 30% loss of olive crops [6]. *B. oleae* leads to olive fruit infestation through chewing damage [7] and pupates either on soil or on olive fruit [8]. Chemical control of *B. oleae* with dimethoate-based insecticides is not always ideal, because it is toxic to bees (*Apis mellifera* L.; Hymenoptera: Apidae) and can be harmful to other organisms [9]. In addition, insecticide residues are often detected in olive oil, which contradicts consumer demand for higher quality [10,11].

Biological control via EPFs in IPM programs could be a regulatory factor of the *B. oleae* population in Greece [10,11]. The concept of IPM programs merges cost-effective management tactics with a low ecological impact to maintain pest populations below damaging levels [12]. EPFs could be useful IPM components in olive cultivation, and a lot of them have been developed for use in IPM programs under field conditions [13] and in greenhouses [14]. EPFs penetrate through the host’s cuticle, colonizing them [15], and they are beneficial for the control of the soil-dwelling life stages of pests [16]. However, there is a lot of unexplored potential regarding EPFs. Many strains could be used as population inhibitors of *B. oleae*, and their study under laboratory conditions may yield noteworthy outcomes. According to Marri et al. (2016) [17], commercial formulations of *Beauveria bassiana* (Hypocreales: Cordycipitaceae) have efficiently controlled invasive *Bactrocera dorsalis* (Diptera: Tephritidae) fruit flies. Also, Wang et al. (2021) [18] observed high mortality of *B. dorsalis* when treated with EPFs in the puparia stage. Moreover, *B. oleae* third-instar pupariation larvae and puparia, according to Youssef et al. (2013) [2], were highly affected by *Metarhizium brunneum* (Hypocreales: Clavicipitaceae), indicating successful mortality. *Metarhizium anisopliae* (Hypocreales: Clavicipitaceae) has been effective in the control management of *B. dorsalis* pupae under laboratory conditions [19]. Pest control of Tephritidae with EPFs is receiving increasing attention, and the next step in research may be to evaluate pest puparium survival, given that soil is the natural ecosystem of EPFs. In certain laboratory experiments, the utilization of soil as an inoculation medium aligns well with real conditions [20,21,22] because it is an important reservoir for a plethora of EPFs [23]. The soil application of *B. bassiana*, *M. anisopliae*, and *Isaria fumosorosea* (Hypocreales: Cordycipitaceae) has been shown to be effective against *Bactrocera zonata* (Diptera: Tephritidae) puparia [24,25].

EPFs are relatively harmless to the environment, with low consequences to beneficial insects [26]. Hence, they could play a more prominent role as pest suppressors in olive culture combined with other management practices in IPM programs, thereby diminishing the use of insecticides [27]. Thus, the IPM of olive cultivation in combination with EPFs may also improve olive oil quality [28]. EPFs as microbial agents remain widely underutilized in Greece’s olive production; simultaneously, steps have been made to implement IPM programs [29]. However, controlling *B. oleae* is very difficult because its appearance is usually observed when damage has already occurred.

In this study, we attempted to unlock more potential in some hard-to-find or widely unexplored soil-inoculated EPFs to limit *B. oleae* pupae as a follow-up to our previous work on the same concept with common EPFs [30]. This study aims to reveal additional insights regarding the biological control of *B. oleae* using EPFs because these microorganisms can be used in IPM programs and are eco-friendly alternatives.

## 2. Materials and Methods

### 2.1. Rearing of Bactrocera oleae

In November 2021, olive fly pupae were collected from oil mills in the Preveza region (Greece) and routinely transferred to the laboratory within 24 h. To obtain same-aged cohorts, the emerged flies were reared in 30 × 30 × 30 cm^3^ net cages in a growth chamber (PHC Europe/Sanyo/Panasonic Biomedical MLR-352-PE) under controlled environmental conditions of 23 ± 2 °C, 65% Relative Humidity (RH), and a 16:8 (L:D) h photoperiod. We reared male and female flies in the same cage and provided a dry diet consisting of sugar and yeast extract (Sigma-Aldrich, Burlington, MA, USA) (4:1) (Sigma-Aldrich, Burlington, MA, USA). Seven days a week, water was replenished on a sponge wick. We sieved sand from the rearing cages three times a day and kept the pupae in small Petri dishes to obtain individuals of the same age. After 4–5 days, they were used for experiments.

### 2.2. EPF Cultures

We obtained seventeen strains of EPFs, belonging to the genera *Aspergillus*, *Fusarium*, *Lecanicillium*, *Penicillium*, *Purpureocillium*, and *Talaromyces* from the personal collection of the first author (Table 1).

### 2.3. Preparation of Fungal Isolates

During 15 days at 25 °C and 65% RH, SDA was used as a medium for cultivating EPF isolates in 9 cm Petri dishes. Petri dishes were sealed with Parafilm^®^ (American National Can, Chicago, IL, USA) to prevent contamination. After 15 days, conidia were collected by scraping the Petri dish surface with a sterile loop and then placed in a 500 mL glass beaker containing 50 mL of sterile distilled water and 0.05% Tween 80 (Sigma-Aldrich, St. Louis, MO, USA). A magnetic stirrer was used to mix the conidial suspension for five minutes after filtering through sterile cloth layers. A Neubauer hemocytometer (Weber Scientific hemocytometer for cell counting, Hamilton Township, NJ, USA) was used to measure the fungal conidium concentration. Dilution was performed by adding 10 mL of the conidial suspension to the required amount of sterile water, resulting in a final concentration of 1 × 10^8^ conidia per ml for the fungal isolates. This specific concentration was chosen due to its widespread use in numerous relevant studies, and conidial viability exceeded 97% for all fungal isolates.

The viability of all the tested fungi was determined by spreading a 100 μL aliquot of a conidial suspension (1 × 10^6^ conidia mL^−1^), prepared with a sterile surfactant solution (0.1% *v*/*v*) of Tween 80, on SDA medium in Petri dishes (90 × 15 mm) and incubated in the dark at 25 ± 1 °C. SDA plates of the tested fungi were incubated for 18 h prior to evaluation. Conidia were scored as viable if any germ tube was 2× longer than the diameter of the spore; a total of 100 conidia per sample were counted under 400× magnification. Conidial viability was calculated based on the formula below:

Viability (%) = [G1/(G1 + G2)] × 100(1)

where G1 refers to the number of germinated conidia, G2 is the number of non-germinated conidia, and the sum of G1 and G2 is equal to 100. Thus, the percentage of viable conidia was determined by counting a total of 100 conidia per fungal sample. Fungal strains presenting ≥95% viability were used in insect bioassays.

### 2.4. The Effect of the Fungal Isolates on the B. oleae Pupae

Seventeen EPF isolates were evaluated against the pupae of *B. oleae*. The collection of pupae was simple, and there was no need to handle them manually: puparia were collected from the lab rearing using a fine brush wet with distilled water. For the first treatment, the bioassay arena was 4–5-day-old pupae buried in the sterilized soil from olive cultivation at a depth of 3 cm, and the second treatment was without soil. A total of 2 mL (1 × 10^8^ conidia mL^−1^) of the solution was sprayed with a 2 mL conidial suspension using a Potter spray tower (Burkard Manufacturing Co., Ltd., Rickmansworth, Hertfordshire, UK) at 1 kgF cm^−2^ onto the surface with soil and without onto the pupae. After mixing, pupae (4–5 days old) were buried individually in cups at a 3 cm depth, and the cups were covered with lids. The control group was sprayed with an aqueous solution with 0.05% Tween 80 (Sigma-Aldrich, St. Louis, MO, USA), which was applied to the soil surface and pupae directly. Pupae that were unable to emerge as adult flies were considered dead. Upon emergence, the adults were transferred to cages (30 cm × 30 cm × 30 cm) and provided with water and adult food, and mortality was recorded over 10 days. Adult mortality and mycosis were determined on a daily basis, and all dead individuals were removed from the cages each day. At each developmental stage (adult or pupa), the individuals were placed inside a plastic Petri dish lined with sterile and moist filter paper (Whatman^®^ Sigma-Aldrich, St. Louis, MO, USA). The dish was wrapped with parafilm^®^ and finally incubated at 25 °C to observe the presence of fungal outgrowth. Before placing them into plastic Petri dishes, pupae and adults were surface sterilized with 1% sodium hypochlorite, followed by three rinses with distilled water. Twenty individuals were used for each treatment replicate. There were ten replicates for each treatment, and the whole experiment was conducted twice, resulting in twenty replications (200 individuals were used for each treatment). The pupa hatch time, mycelium presence on dead pupae, duration from pupation to adult emergence, adult survival time, and mycelium presence on dead adults were determined.

### 2.5. Data Analysis

All values were arcsine transformed prior to analysis. Data were analyzed via two-way ANOVA using the general linear model of SPSS (ΙΒΜ, Inc., Chicago, IL, USA, version 24. In case of significant F values, the means were compared using the Bonferroni test. The Kaplan–Meier method (Life Parameters) was also selected to determine the median lethal time of *B. oleae* following the application of the pathogen concentrations. A comparison of survival distributions was performed using Breslow (Generalized Wilcoxon) (SPSS v.23.0). The male proportion was calculated based on the formula below:

Male Proportion = Male/(Male + Female)

The results from the above formula were expressed as a probability within the range 0–1.

## 3. Results

**Figure 1:**
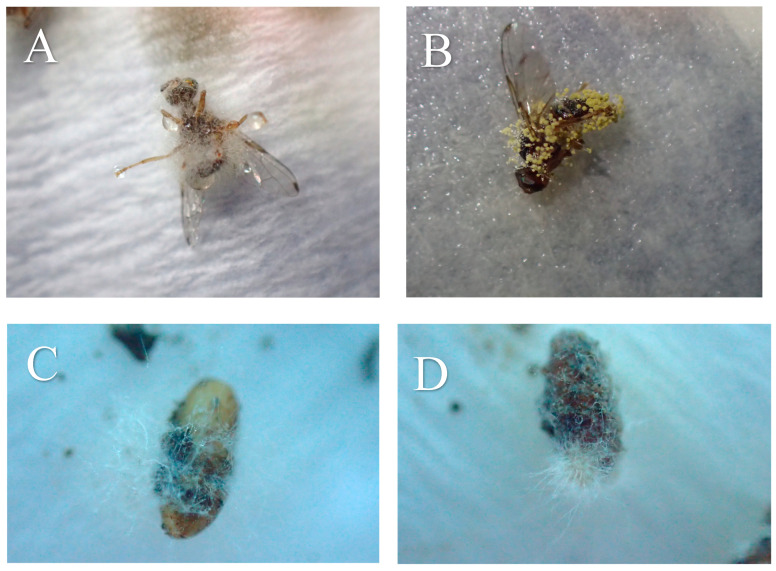
Pupae and hatched adults of *B. oleae* treated with different fungal isolates under laboratory conditions on non-soil: (**A**) *A. austwickii;* (**B**) *A. alliaceus;* (**C**) *F. brachygibbosum;* (**D**) *F. longifundum*.

**Figure 2:**
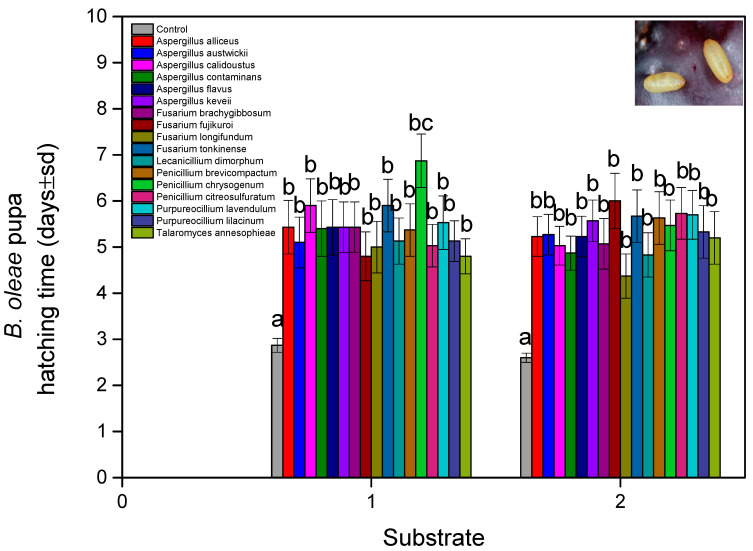
*B. oleae* pupa (days ± sd) hatching time after being treated with fungal isolates in two substrates: (1) soil and (2) non-soil. Different letters between treatments indicate statistically significant differences according to the Bonferroni test.

**Figure 3:**
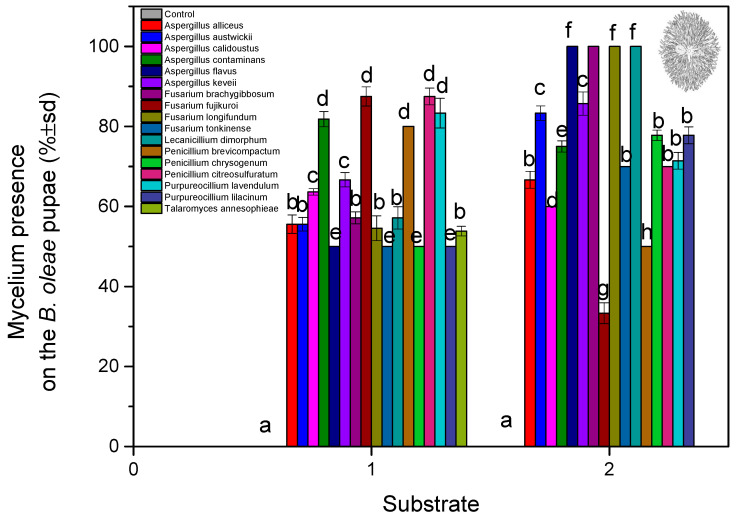
Mycelium (% ± sd) presence on *B. oleae*-treated pupae with fungal isolates in two substrates: (1) soil and (2) non-soil. Different letters between treatments indicate statistically significant differences according to the Bonferroni test.

Accordingly, in relation to the highest Pupa hatch time, this was estimated at 6.87 ± 0.58 days for *P. chrysogenum* (soil) and 5.73 ± 0.56 days for *P. citreosulfuratum* (non-soil). In all other tested isolates, the hatching time was lower than 5.5 days (Figure 1 and Figure 2) (fungal isolates: F = 9.888, df = 16,1620, and *p* < 0.001; exposure time: F = 12.113, df = 9, 1620, and *p* < 0.001; fungal isolates × exposure time: F = 5.743, df = 160, 1620, and *p* < 0.001). The results for the soil substrate showed that external fungal growth was observed in samples treated with *F. fujikuroi–P. citreosulfuratum* (87.5%), *P. lavendulum* (83%), and *A. contaminans* (81.8%). The external fungal growth in other isolations was lower than 70% of the treated pupae. On the other hand, the non-soil vs. substrate showed that external fungal growth was observed in the samples treated with *A. flavus*, *F. longifundum, n* and *L. dimorphum* (100%) (Figure 1 and Figure 3). The external fungal growth in other isolations was lower than 80% of the treated pupae (fungal isolates: F = 11.221, df = 16, 1620, *p* < 0.001; exposure time: F = 10.943, df = 9, 1620, *p* < 0.001; fungal isolates × exposure time F = 7.115, df = 170, 1620, *p* < 0.001). The substrate, as a factor, had an impact on the results: pupa hatch time: F = 15.111, df = 16, 1620, and *p* < 0.001; adult survival time: F = 10.991, df = 16, 1620, and *p* < 0.001; fungal isolates and pupa hatch time × adult survival time × fungal isolates: F = 2.111, df = 4913, 1620, and *p* < 0.001. Mycelium presence on the pupae as a factor had an impact on the results, depending on the substrate that the pupae were in (fungal isolates: F = 4.888, df = 16, 1620, and *p* < 0.001; substrate: F = 2.223, df = 1, 1620, and *p* < 0.001; fungal isolates × exposure time: F = 1.743, df = 16, 1620, and *p* < 0.001).

**Figure 4:**
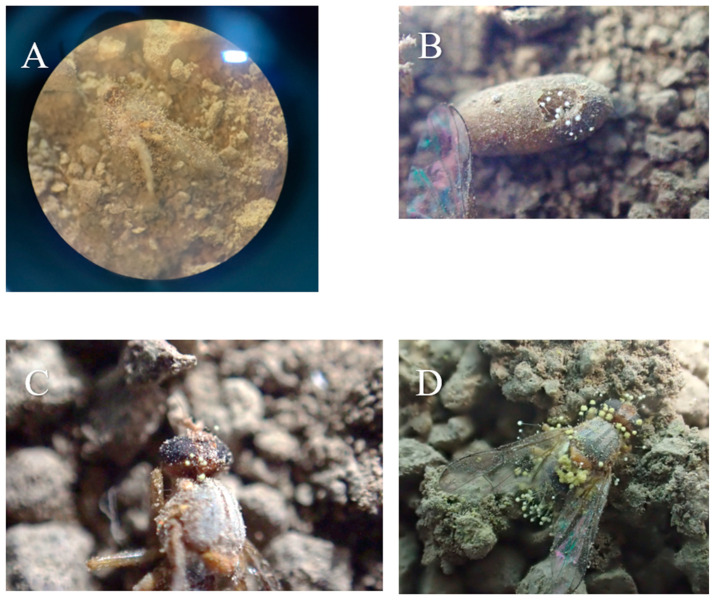
Pupae and hatched adults of *B. oleae* treated with different fungal isolates under laboratory conditions on soil: (**A**) *P. chrysogenum;* (**B**) *A. contaminans;* (**C**) *L. dimorphum;* (**D**) *A. flavus*.

**Figure 5:**
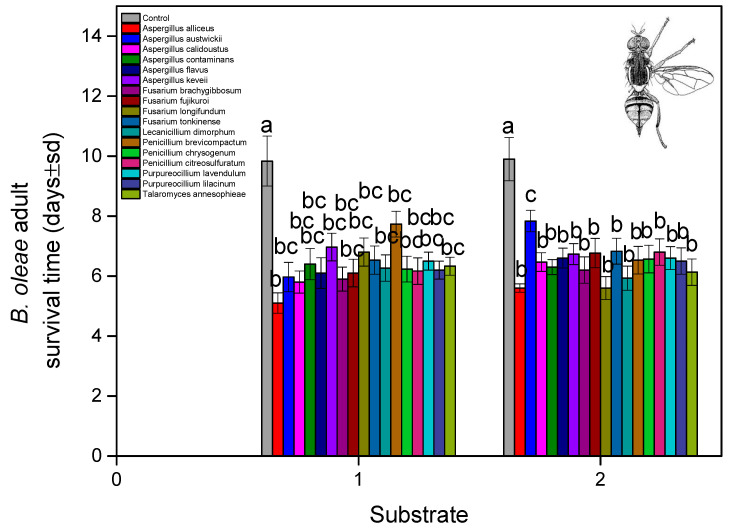
*B. oleae* adult (days ± sd) survival time after treatment with fungal isolates in two substrates: (1) soil and (2) non-soil. Different letters between treatments indicate statistically significant differences according to the Bonferroni test.

Mycelial and conidial growth on cadavers suggested that almost all deaths were pathogen related (Figure 4). The results for the soil substrate showed that external fungal growth was observed in dead adults with *A. contaminans*, *A. keveii*, *A. flavus P. lilacinum*, and *T. annesophieae* (100%), and external fungal growth was not developed enough with *F. tonkinense* (44.44%). The lowest adult survival time was estimated at 5.1 days for *A. alliceus* (soil) and 5.6 ± 0.37 days *A. alliceus* and *F. longifundum* (non-soil). In all other isolations, the median lethal time was over 6 days (Figure 5). (fungal isolates: F = 8.111, df = 16, 1620, and *p* < 0.001; exposure time: F = 15.111, df = 9, 1620, and *p* < 0.001; fungal isolates × exposure time: F = 6.992, df = 160, 1620, and *p* < 0.001).

**Figure 6:**
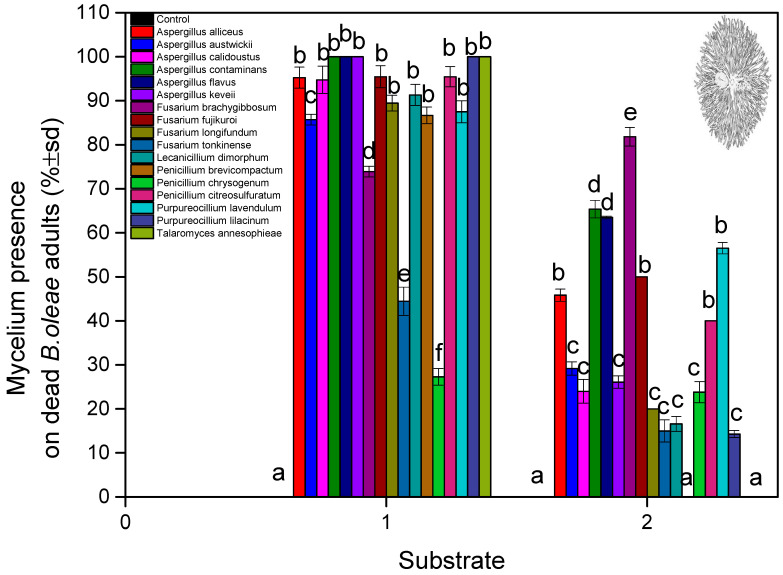
Mycelium (% ± sd) presence on dead *B. oleae* adults after treated pupae with fungal isolates at two substrates: (1) soil and (2) non-soil. Different letters between treatments indicate statistically significant differences according to the Bonferroni test.

On the other hand, with the non-soil substrate, external fungal growth was observed in dead adults with *Fusarium tonkinense* (65.38%), and external fungal growth was not detected in the case of *P. brevicompactum*, and *T. annesophieae* (0%) (fungal isolates: F = 10.345, df = 16, 1620, and *p* < 0.001; exposure time: F = 14.993, df = 9, 1620, and *p* < 0.001; fungal isolates × exposure time: F = 4.875, df = 160, 1620, and *p* < 0.001). The external fungal growth in other isolations was lower than 50% of the treated adults. The control hatch time was 2.87 ± 0.15 days (soil) and 2.60 ± 0.10 days (non-soil) (Figure 6). For the control treatment, no mycelium was found on the pupae or the dead adults. Mycelium presence on the surviving adults as a factor had an impact on the results, depending on the substrate (fungal isolates: F = 3.112 df = 16, 1620, and *p* < 0.001; substrate: F = 2.298, df = 1, 1620, and *p* < 0.001; fungal isolates × substrate: F = 1.659, df = 16, 1620, and *p* < 0.001).

**Figure 7:**
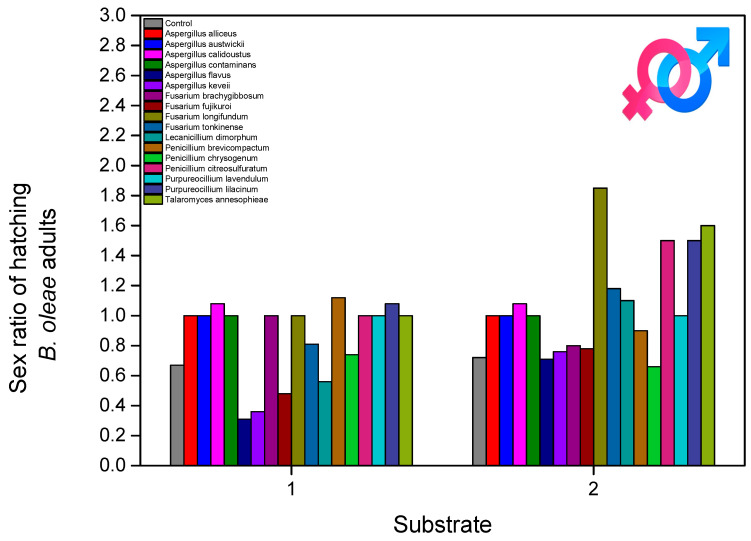
*B. oleae* male proportion after treatment of pupae with fungal isolates in two substrates: (1) soil and (2) non-soil.

The male proportion of the adults hatched from the treated pupae was varied. The male proportion was, for the control, 0.5 for both substrates (Figure 7). For soil and non-soil substrates, the lowest male proportion was for *A. flavus* (0.41–0.42), as well as *A. keveii* (0.36) and *P. citreosulfuratum* (0.41) for soil only. In the case of two *Fusarium* fungi, *F. fujikuroi* (0.48) and *F. tonkinense* (0.47), the male proportion was lower only in the soil treatment. The fungus *T. annesophieae* (0.45) had the same effect on the male proportion in both substrates. The male proportion in the adults that were hatched from the treated pupae had an impact on the results, depending on the fungus isolate (fungal isolates: F = 1.009, df = 16, 1620, and *p* < 0.001; substrate: F = 12.320, df = 1, 1620, and *p* = 0.610; fungal isolates × substrate: F = 9.229, df = 16, 1620, and *p* = 0.022).

**Figure 8:**
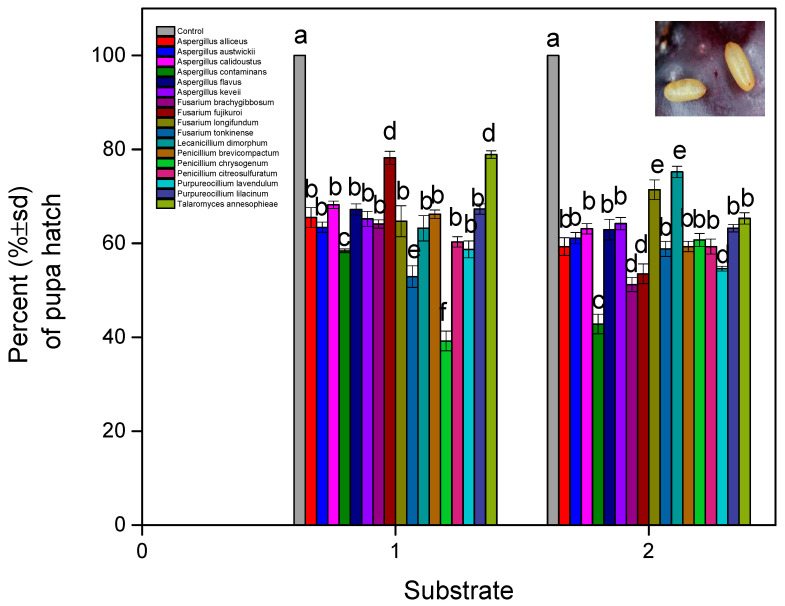
*B. oleae* pupa (% ± sd) hatch after being treated with fungal isolates in two substrates: (1) soil and (2) non-soil. Different letters between treatments indicate statistically significant differences according to the Bonferroni test.

**Figure 9:**
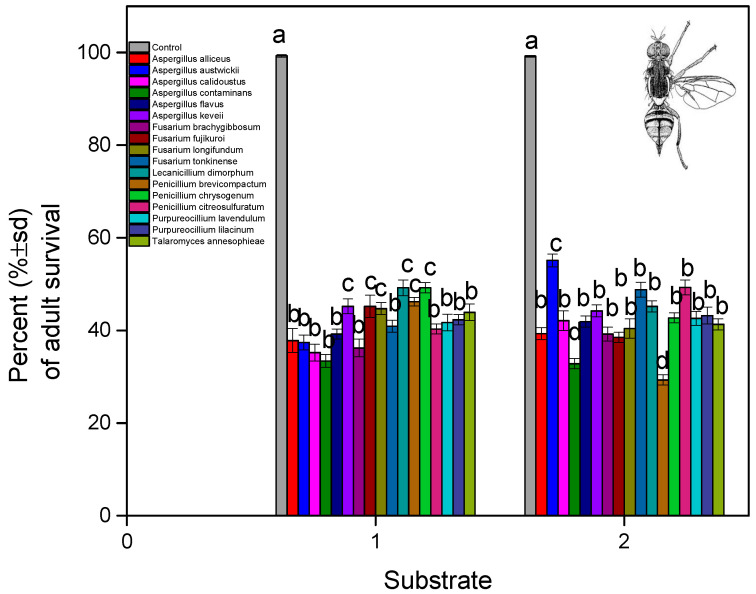
*B. oleae* adult survival (% ± sd) after hatching of the pupae treated with fungal isolates in two substrates: (1) soil and (2) non-soil. Different letters between treatments indicate statistically significant differences according to the Bonferroni test.

Two isolates of *P. chrysogenum* (soil) and *A. contaminans* (non-soil) caused the lowest level of pupa hatching. As expected, the control pupa hatch was very high (100%) (Figure 8). The main effects and interactions for all factors proved to be significant (fungal isolates: F = 5.232, df = 16, 1620, and *p* < 0.001; substrate: F = 12.389, df = 1, 1620, and *p* < 0.001; exposure time: F = 30.844, df = 9, 1620, and *p* < 0.001; fungal isolates × exposure time × substrate: F = 2.435, df = 160, 1620, and *p* < 0.001). This indicates that several fungal isolates affected the survival time of the insect in diverse ways. The survival of the adults was lower with *A. contaminans* (soil) and *P. brevicompactum* (non-soil) (Figure 9). As expected, the control survival was above 99%. The main effects and interactions for all factors proved to be significant (fungal isolates: F = 2.435, df = 16, 1620, and *p* < 0.001; substrate: F = 18.112, df = 1, 1620, and *p* < 0.001; exposure time: F = 30.844, df = 9, and 1620; fungal isolates × exposure time × substrate: F = 2.711, df = 160, 1620, and *p* < 0.001).

## 4. Discussion

Insect EPFs are microbial control agents that play an important role in integrated pest management. These fungi are used as biological control agents for a broad range of insects. The innate immune system of insects includes both cellular and humoral components [31,32,33,34,35]. An epizootic develops depending on several factors, including the host, pathogen, population, and environment [36]. Several processes contribute to the transmission of fungal pathogens: conidial production, discharge, dispersion, survival, and germination [37]. EPFs can release immunosuppressant toxins and produce hyphae in insects despite the host’s immune response. Insecticidal effectiveness is manipulated by many factors, such as insect behavior, population density, age, nutrition, and genetic information. In general, EPFs enter through contact and can penetrate through the insect cuticle, producing hydrolytic enzymes like proteinases, chitinases, and lipases that can infect many Diptera pupae. The highest toxicity (lowest survival time) of the examined EPFs was found on the pupa hatch time of *B. oleae*. The higher pupa hatch time and the higher mycelium presence on these pupae confirm the findings of Bateman et al. (1996) [38], who found that the infection of insects by fungi depends on their biological stage.

De la Rosa et al. (2002) [39] determined that the virulence of each fungus strain depends on the insect from which it was isolated and its susceptibility [37]. Host–pathogen associations may explain the differences between strains or populations of insects [40]. Muñoz et al. (2009) [41] compared 16 strains of *B. bassiana* against *Ceratitis capitata* (Diptera: Tephritidae), reporting mortalities of 12.9 to 91.2% and lethal times of 3.83 to 17.64 days. The lethal times determined by De la Rosa et al. (2002) [39] ranged from 2.82 to 5.99 days when they evaluated seven strains of *B. bassiana* against *Anastrepha ludens* (Diptera: Tephritidae). According to Lezama-Gutiérrez et al. (2000) [42], the mortalities exceeded 83.7% when using *M. anisopliae* against *A. ludens*, and Hernández-Díaz-Ordaz et al. (2010) [43] reported the two strains of *B. bassiana* and one of *M. anisopliae* against adults of *A. obliqua*. Osorio-Fajardo and Canal (2011) [44] reported on two strains of *B. bassiana* and one of *M. anisopliae,* recording lethal times of 42.7, 48.1, and 56 h, respectively, against *A. obliqua*. Quesada-Moraga et al. (2006) [45] reported LC_50_ values against *C. capitata* with *B. bassiana.* The highest toxicity (lowest survival time) of the examined EPFs was found for *B. oleae* adults. Similar results were obtained by Konstantopoulou and Mazomenos (2005) [46], who, in evaluating different EPFs against adults of *B. oleae*, obtained lower survival times for the treated adults after 14 days of treatment.

Low levels of mortality in pupae caused by EPFs have been documented in various studies against *B. zonata* (Diptera: Tephritidae) [25,47,48]. Such variations among various EPF strains of the same species in the same host have been well documented in many relevant assays. Hussein et al. (2018) [48] observed higher adult emergence of *B. zonata* from 4-day-old pupae compared to 1-day-old pupae when treated with *B. bassiana* and *M. anisopliae*. The results reported by Beris et al. (2013) [49] and Furlong and Pell (2001) [50] used different EPF isolates, insect species, and application rates. The low susceptibility of pupae recorded in this study may be due to the use of older pupae (4–5 days old). Ekesi et al. (2002) [51] found that the pupal susceptibility to *M. anisopliae* was reduced with an increased age of pupae of *C. capitata*. The reason behind the high susceptibility of younger pupae to fungal infection seems to be due to the softer cuticles of young pupae [51]. Interestingly, high adult mortality was recorded after emergence from the treated pupae in this study. These results agree with the findings from other studies showing high adult mortality from infected pupae in *B. oleae* and insect pests of other species [51,52,53]. High levels of *A. ludens* adult mortality were observed when old pupae (2 days before adult emergence) were treated [54]. Soil serves as a natural ecosystem for EPFs, providing fungi with optimal moisture and temperature conditions and protection against UV radiation [55]. More importantly, soil also serves as a habitat where EPFs come into contact with the soil-dwelling life stages of insects. Consequently, the persistence of EPFs in the soil is a requirement for successful control. Garrido-Jurado et al. (2011) [56] reported that the availability of *B. bassiana* and *M. anisopliae* is significantly affected by soil properties, although no significant effects were recorded on the pathogenicity of EPFs. Since the rate of fungal movement through the soil profile is low, most of the available spores are retained within the superficial soil layer and persist within the roots and insects after soil application [56,57]. Considering that most *Tuta absoluta* (Lepidoptera: Gelechiidae) individuals pupate in soil at a depth of 1–2 cm [58], there is a high probability that pupae will encounter conidia of EPFs if applied as drench treatments. These results agree with our findings that the presence of soil helps the pathogenicity or transmission of conidia. A logical explanation for this outcome is that soil provides a microhabitat that protects conidia from desiccation, light, and extreme temperatures, enhancing their viability and pathogenicity. Moreover, soil particles adhere to conidia, improving their attachment to insect cuticles and facilitating direct contact.

Several studies have confirmed the ability of EPFs to be transmitted horizontally. Examples include *A. ludens* [59], *C. capitata* [49,60], *B. zonata*, *Bactrocera cucurbitae* (Diptera: Tephritidae) [61], and other insect species, such as *Anopheles gambiae* (Diptera: Culicidae) [62] and *Glossina morsitans* (Diptera: Glossinidae) [63]. All previous studies had shown significant mortality (e.g., 85–100% in *C. capitata* [2], 69–83% in *B. zonata*, and 78–88% in *B. cucurbitae* [61]). In our study, infected males of both species were highly infectious to females. Quesada-Moraga et al. (2008) [60] observed that males of *C. capitata* were able to disseminate more conidia to females compared to female-to-male transmission. This may be related to the mating process. Sookar et al. (2014) [61] suggested that the females disseminated more conidia to the males compared to males to females against *B. zonata* and *B. cucurbitae* in different pairing combinations. Various physiological characteristics of insects, including their age, sex, and nutritional status, can be influenced by their susceptibility to fungal infections. Infection with fungi strongly reduced, from our results, the proportion of *B. oleae* males. Remarkably, the male proportion recorded after emergence from treated pupae in this study was lower for *A. flavus* in both substrates. To our knowledge, this is the first study to report the effect of *A. flavus* on the male proportion of *B. oleae*.

The moderate to high level of virulence we observed among fungi to *B. oleae* pupae is consistent with some other studies. Beris et al. 2013 [49] reported low susceptibility of the Mediterranean fruit fly, *C. capitata* (Wiedemann), pupae when exposed to different fungal species at the same concentration that we used. In our case, the pupa hatch time was estimated at 6.9 days for *P. chrysogenum* (soil) and 5.7 days *P. citreosulfuratum* (non-soil). In all other isolates, the median lethal time was lower, 5.5 days, and with the control, 2.9 days.

## 5. Conclusions

This study aims to contribute toward filling the research gaps of some less mainstream EPF effects on *B. oleae* pupae. The high efficacy of some EPFs on the pupa hatch time and mycelium presence on dead *B. oleae* pupae and adults, especially of *A. contaminans*, *A. flavus*, and *A. keveii,* prompts us to explore further the prospects of testing many of these strains in IPM programs for the protection of olive groves from *B. oleae*, which significantly reduces the quality and quantity of olive oil in Greece. Our results suggest that we can consider the possibility of using biological control with EPFs as an alternative strategy to control *B. oleae* in Europe. Studies that examine the isolates under field conditions are needed to further evaluate the fungi as potential control agents for the fly. Although the experiment was laboratory conducted, the conditions were realistically compensated, as the survival of the EPFs on *B. oleae* pupae was carried out in real olive grove soil samples from areas with economically important olive oil exports. Concerning the commercialization and registration of future isolates, the following question remains: what further rules and testing are required to provide the user and customer with a safe biocontrol product? Under this spectrum, further trials should be conducted under various climatic conditions, testing additional fungal isolates, or exploring combinations of EPFs with other biocontrol agents that could enhance the effectiveness and applicability of *B. oleae* biocontrol strategies

## Figures and Tables

**Table 1:** EPF species that were tested in the present study.

Fungus Species	Isolate	Collection Site
*Aspergillus alliaceus*	1	Kastritsi Achaia
*Aspergillus austwickii*	2	Dasyllio Achaia
*Aspergillus calidoustus*	3	Dasyllio Achaia
*Aspergillus contaminans*	4	Dasyllio Achaia
*Aspergillus flavus*	6	Dasyllio Achaia
*Aspergillus keveii*	10	Elos Achaia
*Fusarium brachygibbosum*	19	Elos Achaia
*Fusarium fujikuroi*	20	Elos Achaia
*Fusarium longifundum*	21	Elos Achaia
*Fusarium tonkinense*	22	Dasyllio Achaia
*Lecanicillium dimorphum*	23	Dasyllio Achaia
*Penicillium brevicompactum*	25	Elos Achaia
*Penicillium chrysogenum*	26	Dasyllio Achaia
*Penicillium citreosulfuratum*	27	Dasyllio Achaia
*Purpureocillium lavendulum*	37	Elos Achaia
*Purpureocillium lilacinum*	38	Dasyllio Achaia
*Talaromyces annesophieae*	40	Elos Achaia

## Data Availability

The data presented in this study are available on request from the corresponding author S.M.

## References

[1] Gonçalves M.F., Malheiro R., Casal S., Torres L., Pereira J.A. (2012). Influence of Fruit Traits on Oviposition Preference of the Olive Fly, *Bactrocera oleae* (Rossi) (Diptera: Tephritidae), on Three Portuguese Olive Varieties (Cobrançosa, Madural and Verdeal Transmontana). Sci. Hortic..

[2] Yousef M., Lozano-Tovar M.D., Garrido-Jurado I., Quesada-Moraga E. (2013). Biocontrol of *Bactrocera oleae* (Diptera: Tephritidae) with *Metarhizium brunneum* and Its Extracts. J. Econ. Entomol..

[3] Gomez J.A., Amato M., Celano G., Koubouris G.C. (2008). Organic Olive Orchards on Sloping Land: More than a Specialty Niche Production System?. J. Environ. Manag..

[4] Michalopoulos G., Kasapi K.A., Koubouris G., Psarras G., Arampatzis G., Hatzigiannakis E., Kavvadias V., Xiloyannis C., Montanaro G., Malliaraki S. (2020). Adaptation of Mediterranean Olive Groves to Climate Change through Sustainable Cultivation Practices. Climate.

[5] Skiada V., Tsarouhas P., Varzakas T. (2019). Preliminary Study and Observation of “Kalamata PDO” Extra Virgin Olive Oil, in the Messinia Region, Southwest of Peloponnese (Greece). Foods.

[6] Athar M. (2005). Infestation of Olive Fruit Fly, *Bactrocera oleae*, in California and Taxonomy of Its Host Trees. Agric. Conspec. Sci..

[7] Valenčič V., Butinar B., Podgornik M., Bučar-Miklavčič M. (2020). The Effect of Olive Fruit Fly *Bactrocera oleae* (Rossi) Infestation on Certain Chemical Parameters of Produced Olive Oils. Molecules.

[8] Preu M., Frieß J.L., Breckling B., Schröder W. (2020). Case Study 1: Olive Fruit Fly (*Bactrocera oleae*). Gene Drives at Tipping Points: Precautionary Technology Assessment and Governance of New Approaches to Genetically Modify Animal and Plant Populations.

[9] Pontikakos C.M., Tsiligiridis T.A., Yialouris C.P., Kontodimas D.C. (2012). Pest Management Control of Olive Fruit Fly (*Bactrocera oleae*) Based on a Location-Aware Agro-Environmental System. Comput. Electron. Agric..

[10] Balampekou E.I., Koutsos T.M., Menexes G.C., Koveos D.S., Kouloussis N.A. (2024). Pest Management Pathways: Control Strategies for the Olive Fruit Fly (*Bactrocera oleae*)—A Systematic Map. Agronomy.

[11] Katsikogiannis G., Kavroudakis D., Tscheulin T., Kizos T. (2023). Population Dynamics of the Olive Fly, *Bactrocera oleae* (Diptera: Tephritidae), Are Influenced by Different Climates, Seasons, and Pest Management. Sustainability.

[12] Khan S.A., Malik S.H., Lone I.U., Bhat D.M. (2024). Eco-Friendly Pest Management Strategies for Conservation of Biodiversity. Insect Divers. Ecosyst. Serv..

[13] Priyashantha A.K.H., Galappaththi M.C.A., Karunarathna S.C., Lumyong S. (2024). Entomopathogenic Fungi: Bioweapons against Insect Pests. The Role of Entomopathogenic Fungi in Agriculture.

[14] Gonçalves R.B., Aparecida M., Zawadneak C., Melissa De Azevedo Kuhn T., Fernando T., Gulinelli M., Pimentel I.C., Poltronieri A.S., Machado Da Rosa J., Mirás-Avalos J.M. (2025). Occurrence and Behavior Analysis of *Duponchelia fovealis* on Strawberry Plants: Insights for Integrated Pest Management. Horticulturae.

[15] Hong S., Shang J., Sun Y., Tang G., Wang C. (2024). Fungal Infection of Insects: Molecular Insights and Prospects. Trends Microbiol..

[16] Brunner M., Zeisler C., Neu D., Rotondo C., Rubbmark O.R., Reinbacher L., Grabenweger G., Traugott M. (2024). Trap Crops Enhance the Control Efficacy of *Metarhizium brunneum* against a Soil-Dwelling Pest. J. Pest Sci..

[17] Marri D., Gomez D.A.M.A., Wilson D.D., Billah M., Yeboah S., Osae M. (2016). Evaluation of the Efficacy of a Commercial Formulation of *Beauveria bassiana* for the Control of the Invasive Fruit Fly *Bactrocera dorsalis* (Diptera: Tephritidae). Biopestic. Int..

[18] Wang D., Liang Q., Chen M., Ye H., Liao Y., Yin J., Lü L., Lei Y., Cai D., Jaleel W. (2021). Susceptibility of Oriental Fruit Fly, *Bactrocera dorsalis* (Diptera: Tephritidae) Pupae to Entomopathogenic Fungi. Appl. Entomol. Zool..

[19] Abdellah A.M., Hassan A.E.M., Eisa A.A., Adam Y.S., Dafallah A.B., Abdellah A.G.M., Hassan A.E.M., Eisa A.A., Dafallah A.B., Adam Y.S. (2020). Efficacy of a Sudanese Strain of Entomopathogenic Fungus, *Metarhizium anisopliae* Met. Sorokin on Puparia of *Bactrocera dorsalis* Hendel, Under Laboratory Conditions. Sustain. Manag. Invasive Pests Afr..

[20] Mantzoukas S., Eliopoulos P.A. (2020). Endophytic Entomopathogenic Fungi: A Valuable Biological Control Tool against Plant Pests. Appl. Sci..

[21] Sani I., Jamian S., Ismail S.I., Saad N., Abdullah S., Hata E.M., Kamarudin M.A., Jalinas J. (2022). Identification of Entomopathogenic Fungi *Metarhizium anisopliae* and *Purpureocillium lilacinum* from Oil Palm Plantation Soils in Universiti Putra Malaysia. Malays. J. Microbiol..

[22] Sani I., Jamian S., Saad N., Abdullah S., Hata E.M., Jalinas J., Ismail S.I. (2023). Inoculation and Colonization of the Entomopathogenic Fungi, *Isaria javanica* and *Purpureocillium lilacinum*, in Tomato Plants, and Their Effect on Seedling Growth, Mortality and Adult Emergence of Bemisia Tabaci (Gennadius). PLoS ONE.

[23] Majchrowska-Safaryan A., Tkaczuk C. (2021). Abundance of Entomopathogenic Fungi in Leaf Litter and Soil Layers in Forested Habitats in Poland. Insects.

[24] Murtaza G., Naeem M., Manzoor S., Khan H.A., Eed E.M., Majeed W., Makki H.A., Ramzan U., Ummara U.E. (2022). Biological Control Potential of Entomopathogenic Fungal Strains against Peach Fruit Fly, *Bactrocera zonata* (Saunders) (Diptera: Tephritidae). PeerJ.

[25] Tahira Gul H., Freed S., Akmal M., Naeem Malik M. (2015). Vulnerability of Different Life Stages of *Bactrocera zonata* (Tephritidae: Diptera) Against Entomogenous Fungi. Pak. J. Zool..

[26] Saminathan N., Subramanian J., Sankaran Pagalahalli S., Theerthagiri A., Mariappan P. (2024). Entomopathogenic Fungi: Translating Research into Field Applications for Crop Protection. Arthropod-Plant Interact..

[27] El Aalaoui M., Rammali S., Bencharki B., Sbaghi M. (2025). Efficacy of Biorational Insecticides and Entomopathogenic Fungi for Controlling *Cassida vittata* Vill. (Coleoptera: Chrysomelidae) in Sugar Beet Crops. Neotrop. Entomol..

[28] Intonti M., Mola D., De Leonardis M., Starace G. (2025). Enhancing Circular Practices in Olive Oil Production: The Role of Green Finance. Sustainability.

[29] Anagnostopoulos C., Vidali E., Zacharia E., Dris S. (2025). Evaluation of Terminal Residues in Olives and Olive Oil Following a Targeted Large-Scale Plant Protection Application Strategy in Southern Greece. J. Food Compos. Anal..

[30] Mantzoukas S., Margaritis A., Sourouni T., Georgopoulou V., Zarmakoupi C., Papantzikos V., Lagogiannis I., Eliopoulos P.A., Patakioutas G. (2024). Insecticidal Action of Local Isolates of Entomopathogenic Fungi Against *Bactrocera oleae* Pupae. Biology.

[31] Rosengaus R.B., Traniello J.F.A., Chen T., Brown J.J., Karp R.D., Rosengaus R.B., Traniello J.F.A., Chen T., Brown J.J., Karp R.D. (1999). Immunity in a Social Insect. Naturwissenschaften.

[32] Lamberty M., Zachary D., Lanot R., Bordereau C., Robert A., Hoffmann J.A., Bulet P. (2001). Insect Immunity. Constitutive Expression of a Cysteine-Rich Antifungal and a Linear Antibacterial Peptide in a Termite Insect. J. Biol. Chem..

[33] Cox F.E.G. (2001). Concomitant Infections, Parasites and Immune Responses. Parasitology.

[34] Barton N.H., Briggs D.E.G., Eisen J.A., Goldstein D.B., Patel N.H. (2007). Evolution.

[35] Graham A.L. (2008). Ecological Rules Governing Helminth-Microparasite Coinfection. Proc. Natl. Acad. Sci. USA.

[36] Tanada Y., Kaya H.K. (1993). Insect Pathology.

[37] Hajek A.E., St. Leger R.J. (1994). Interactions between Fungal Pathogens and Insect Hosts. Annu. Rev. Entomol..

[38] Bateman R., Carey M., Batt D., Prior C., Abraham Y., Moore D., Jenkins N., Fenlon J. (1996). Screening for Virulent Isolates of Entomopathogenic Fungi Against the Desert Locust, *Schistocerca gregaria* (Forskål). Biocontrol Sci. Technol..

[39] De la Rosa W., Lopez F.L., Liedo P. (2002). *Beauveria bassiana* as a Pathogen of the Mexican Fruit Fly (Diptera: Tephritidae) under Laboratory Conditions. J. Econ. Entomol..

[40] Lecuona R. (1996). Microorganismos Patógenos Empleados En El Control Microbiano de Insectos Plaga.

[41] Muñoz J.A., la Rosa W.D., Toledo J. (2009). Mortalidad En Ceratitis Capitata (Wiedemann) (Diptera: Tephritidae) Por Diversas Cepas de *Beauveria bassiana* (Bals.) Vuillemin, En Condiciones de Laboratorio. Acta Zoológica Mex. (N.S.).

[42] Lezama-Gutiérrez R., De La Luz A.T., Molina-Ochoa J., Rebolledo-Dominguez O., Pescador A.R., López-Edwards M., Aluja M. (2000). Virulence of *Metarhizium anisopliae* (Deuteromycotina: Hyphomycetes) on Anastrepha Ludens (Diptera: Tephritidae): Laboratory and Field Trials. J. Econ. Entomol..

[43] Díaz-Ordaz N.H., Pérez N., Toledo J. (2010). Patogenicidad de Tres Cepas de Hongos Entomopatógenos a Adultos de Anastrepha Obliqua (Macquart) (Diptera: Tephritidae) En Condiciones de Laboratorio. Acta Zoológica Mex. (N.S.).

[44] Osorio-Fajardo A., Canal N.A. (2011). Selección de Cepas de Hongos Entomopatógenos Para El Manejo de Anastrepha Obliqua (Macquart, 1835) (Diptera: Tephritidae) En Colombia. Rev. Fac. Nac. Agron. Medellin.

[45] Quesada-Moraga E., Ruiz-García A., Santiago-Álvarez C. (2006). Laboratory Evaluation of Entomopathogenic Fungi *Beauveria bassiana* and *Metarhizium anisopliae* against Puparia and Adults of Ceratitis Capitata (Diptera: Tephritidae). J. Econ. Entomol..

[46] Konstantopoulou M.A., Mazomenos B.E. (2005). Evaluation of *Beauveria bassiana* and *B. brongniartii* Strains and Four Wild-Type Fungal Species against Adults of *Bactrocera oleae* and *Ceratitis capitata*. BioControl.

[47] Farag Mahmoud M. (2009). Susceptibility of the Peach Fruit Fly, *Bactrocera zonata* (Saunders), (Diptera: Tephritidae) to Three Entomopathogenic Fungi. Egypt. J. Biol. Pest Control.

[48] Hussein M.A., Khaled A.S., Ibrahim A.A., Soliman N.A., Attia S.H. (2018). Evaluation of Entomopathogenic Fungi, Beauveriabassiana and *Metarhizium anisopliae* on Peach Fruit Fly, *Bactrocera zonata* (Saunders) (Diptera:Tephritidae). Egypt. Acad. J. Biol. Sci. F. Toxicol. Pest Control.

[49] Beris E.I., Papachristos D.P., Fytrou A., Antonatos S.A., Kontodimas D.C. (2013). Pathogenicity of Three Entomopathogenic Fungi on Pupae and Adults of the Mediterranean Fruit Fly, Ceratitis Capitata (Diptera: Tephritidae). J. Pest Sci..

[50] Furlong M.J., Pell J.K. (2001). Horizontal Transmission of Entomopathogenic Fungi by the Diamondback Moth. Biol. Control.

[51] Ekesi S., Maniania N.K., Lux S.A. (2002). Mortality in Three African Tephritid Fruit Fly Puparia and Adults Caused by the Entomopathogenic Fungi, *Metarhizium anisopliae* and *Beauveria bassiana*. Biocontrol Sci. Technol..

[52] Poprawski T.J., Robert P.H., Majchrowicz I., Boivin G. (1985). Susceptibility of *Delia antiqua* (Diptera: Anthomyiidae) to Eleven Isolates of Entomopathogenic Hyphomycetes. Environ. Entomol..

[53] Goble T.A., Dames J.F., Hill M.P., Moore S.D. (2011). Investigation of Native Isolates of Entomopathogenic Fungi for the Biological Control of Three Citrus Pests. Biocontrol Sci. Technol..

[54] Ekesi S., Maniania N.K., Mohamed S.A., Lux S.A. (2005). Effect of Soil Application of Different Formulations of *Metarhizium anisopliae* on African Tephritid Fruit Flies and Their Associated Endoparasitoids. Biol. Control.

[55] Jaronski S.T. (2010). Ecological Factors in the Inundative Use of Fungal Entomopathogens. BioControl.

[56] Garrido-Jurado I., Torrent J., Barrón V., Corpas A., Quesada-Moraga E. (2011). Soil Properties Affect the Availability, Movement, and Virulence of Entomopathogenic Fungi Conidia against Puparia of *Ceratitis capitata* (Diptera: Tephritidae). Biol. Control.

[57] Ignoffo C.M., Garcia C., Hostetter D.L., Pinnell R.E. (1977). Vertical Movement of Conidia of *Nomuraea rileyi* Through Sand and Loam Soils. J. Econ. Entomol..

[58] Uchoa-Fernandes M.A., Della Lucia T.M.C., Vilela E.F. (1995). Anais Da Sociedade Entomológica Do Brasil. An. Soc. Entomológica Bras..

[59] Ekesi S., Maniania N.K., Lux S.A. (2003). Effect of Soil Temperature and Moisture on Survival and Infectivity of *Metarhizium anisopliae* to Four Tephritid Fruit Fly Puparia. J. Invertebr. Pathol..

[60] Quesada-Moraga E., Martin-Carballo I., Garrido-Jurado I., Santiago-Álvarez C. (2008). Horizontal Transmission of *Metarhizium anisopliae* among Laboratory Populations of *Ceratitis capitata* (Wiedemann) (Diptera: Tephritidae). Biol. Control.

[61] Sookar P., Bhagwant S., Allymamod M.N. (2014). Effect of *Metarhizium anisopliae* on the Fertility and Fecundity of Two Species of Fruit Flies and Horizontal Transmission of Mycotic Infection. J. Insect Sci..

[62] Scholte E.J., Knols B.G.J., Takken W. (2004). Autodissemination of the Entomopathogenic Fungus *Metarhizium anisopliae* amongst Adults of the Malaria Vector *Anopheles gambiae s.s.*. Malar. J..

[63] Kaaya G.P., Okech M.A. (1990). Horizontal Transmission of Mycotic Infection in Adult Tsetse, *Glossina morsitans morsitans*. Entomophaga.

